# The spatiotemporal development of the midlatitude troughs and subauroral ion drift during a geomagnetic storm observed by multiple DMSP satellites

**DOI:** 10.1186/s40623-025-02349-9

**Published:** 2025-12-24

**Authors:** Heechan Cha, Jerry Goldstein, Dhirendra Kataria, Yukitoshi Nishimura, Keiichi Ogasawara

**Affiliations:** 1 Department of Physics and Astronomy, The University of Texas at San Antonio, 1 UTSA circle, San Antonio, TX 78249 USA; 2 Space Science and Engineering Division, Southwest Research Institute, San Antonio, TX 78238 USA; 3 Department of Electrical and Computer Engineering and Center for Space Physics, Boston University, Boston, MA 02215 USA

**Keywords:** Midlatitude trough, Subauroral ion drifts, Magnetosphere–ionosphere coupling

## Abstract

**Abstract:**

Subauroral ion drift (SAID) is a narrow and rapid westward ion flow observed in the subauroral ionosphere during geomagnetic storms and substorms. It is more localized and intense than subauroral polarization streams (SAPS), typically appearing equatorward of auroral boundaries and often associated with midlatitude troughs. This study analyzes ion drifts and plasma density variations using DMSP F16, F17, and F18 data from June 1, 2013, in the Southern Hemisphere. Using multi-satellite observations from three DMSP spacecraft, we systematically examine the spatiotemporal evolution of a SAID event and its associated midlatitude troughs, focusing on their relation to geomagnetic storm phases and substorm activity. We develop an ad hoc empirical model that reproduces SAID spatial distribution and temporal evolution by establishing a quantitative relationship between SAID velocity and the AE index. From the results, we present two key findings: first, we identified a previously unreported two-stage development pattern of SAID: equatorward expansion with minimal width change and moderate potential drop in the early main phase, followed by latitudinal stabilization, width variation, and stronger electric fields in the late main phase. Second, we newly identified that the midlatitude trough developed through three distinct stages: mild density gradient associated with the initial AE increase, sharp density drop at the plasmapause boundary after the first AE decrease, and persistent deep trough after first AE peak and throughout the second AE peak lasted for three hours. These findings and our empirical modeling approach provide new quantitative insights into the distinct temporal evolution patterns of SAID and midlatitude troughs, advancing further understanding of the connection between ionospheric disturbances and geomagnetic storms.

**Graphical Abstract:**

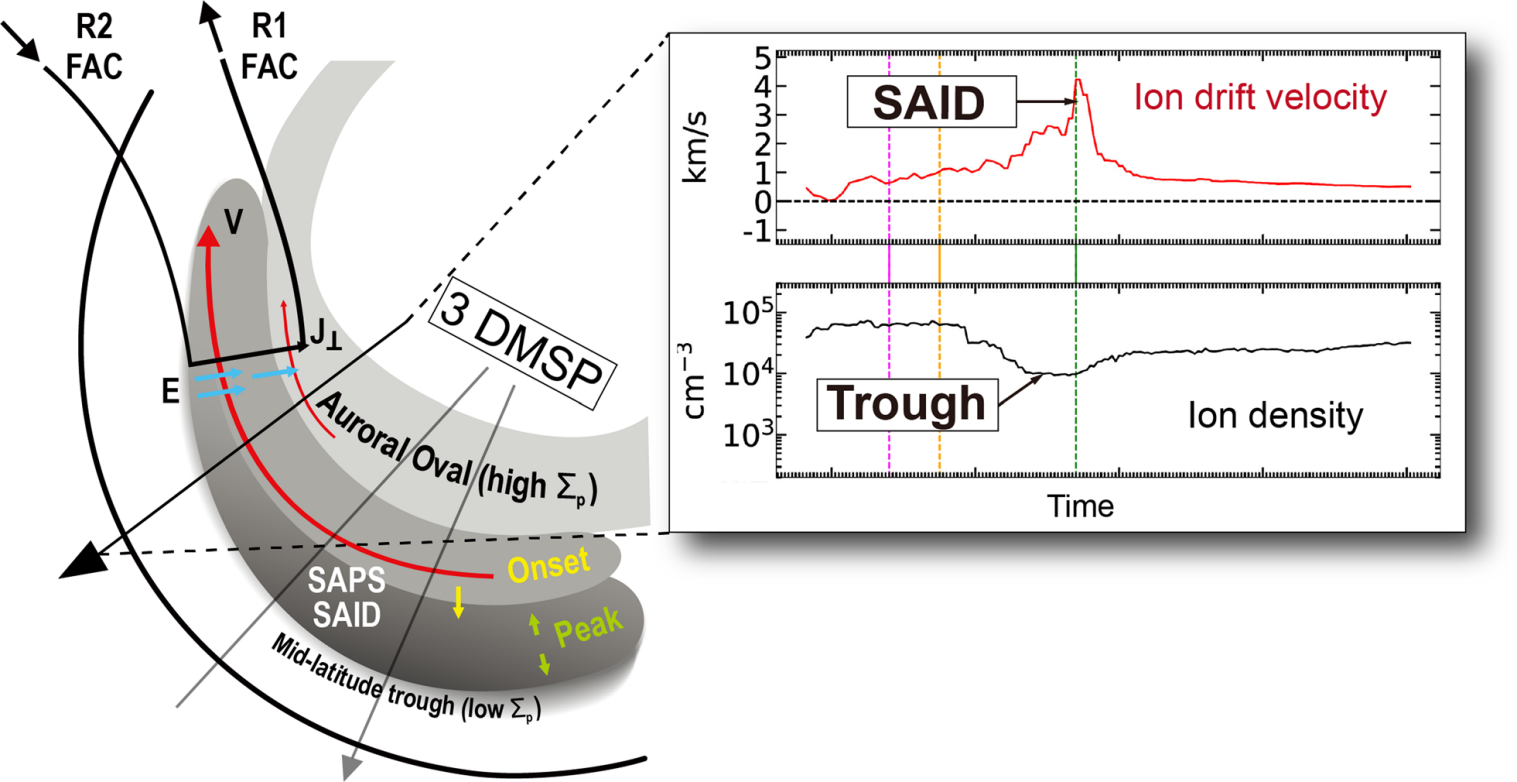

## Introduction

Subauroral ion drift (SAID) is a rapid and narrow channel of westward ion flow observed in the subauroral ionosphere and magnetosphere, during geomagnetic storms and substorms (Foster and Burke 2002). SAID is often described as a more localized and intense version of subauroral polarization streams (SAPS), with drift speeds exceeding 1 km/s and widths less than 2$^{\circ }$ in magnetic latitude (MLAT) (Anderson et al. 1993, 2001; Karlsson et al. 1998; He et al. 2014). Statistically, SAID is formed between 55$^{\circ }$ and 65$^{\circ }$ MLAT and between 18 and 24 h in magnetic local time (MLT), equatorward of the auroral electron precipitation boundary (Karlsson et al. 1998; He et al. 2014). SAID also tends to be narrower in the midnight sector than in the dusk sector (Anderson et al. 2001). Double-peak subauroral ion drift (DSAID) is characterized as flow channels separated from a single-peak SAID flow channel (He et al. 2016). SAID is often associated with a midlatitude trough, a region of reduced ionospheric plasma density, particularly under enhanced geomagnetic conditions (Anderson et al. 2001; He et al. 2012; Heilig et al. 2022). The density depletion in midlatitude trough is caused by increased charge exchange reaction rates due to rapidly moving ions in the flow channels (Schunk et al. 1976; Anderson et al. 1993).

SAID develops through both external (e.g., upstream conditions) and internal (e.g., magnetospheric drivers) processes. The formation and evolution of SAID and the midlatitude trough occur sequentially under active magnetospheric conditions, indicating a strong coupling between magnetospheric drivers and ionospheric particles in the subauroral region (Anderson et al. 1991; Karlsson et al. 1998; Foster and Vo 2002). This coupling is particularly evident during geomagnetic substorms; while the energy injection during a substorm’s expansion phase, marked by a peak in the auroral electrojet (AE) index, enhances the Region 2 field-aligned currents (R2 FACs), the resulting onset and intensification of SAID typically occurs with a time delay during the subsequent recovery phase (Karlsson et al. 1998; Nishimura et al. 2020). The AE index reflects high-latitude magnetic field perturbation caused by the auroral activities especially those associated with a geomagnetic substorm. Similarly, the Dst index tracks storm-time ring current intensity; it delineates storm phases (initial, main, and recovery) that are associated with distinct SAID characteristics. During geomagnetic disturbances, the separation between ion and electron boundaries in the plasma sheet drives the R2 FACs and polarization electric fields essential for SAID formation. These R2 FACs flow into areas of low conductivity in the subauroral ionosphere (Foster and Burke 2002; Anderson et al. 1993). This current system generates an intense poleward electric field that drives the characteristic westward plasma flows in SAID.

Identifying the formation and evolution of SAID has been challenging due to the relatively long orbital period ($\sim$100 min) of low-Earth orbit (LEO) satellites compared to the typical duration of SAID, which ranges from about 30 min to 3 h (Anderson et al. 1991; Lejosne and Mozer 2017). A single polar-orbiting LEO spacecraft passing through a SAID event may sample its time-changing latitudinal profile only once or twice before it dissipates. The rapid transit of LEO satellites through the subauroral region, typically in just a few minutes, provides only a snapshot of its structure. By the time a satellite returns to the same region after completing a full orbit, the SAID would have altered its location, strength, or latitudinal extent. Moreover, such a latitudinal slice by a single satellite cannot quantify the local-time extent of SAID or capture the simultaneous formation of multiple flow channels during complex storm conditions. While ground-based observations, particularly SuperDARN radars, can provide insights into the spatiotemporal characteristics of subauroral fast flows (Kunduri et al. 2017, 2018; Nishitani et al. 2019), multi-satellite in situ observations are essential for providing high-resolution ground-truth measurements and resolving the vertical structure of these phenomena. Furthermore, although the relationship between flow channel intensity and associated trough depth has been measured in individual STEVE events (Nishimura et al. 2020), the systematic investigation of how this relationship varies across different storm phases and responds to changing geomagnetic conditions remains incomplete.

This study examines the spatial and temporal features of one SAID event characterized by two flow channels and two accompanying midlatitude troughs using multi-point observations from three DMSP satellites across different storm phases on 1 June 2013. Building on established multi-satellite methodologies (Anderson et al. 2001; Mishin et al. 2017), we advance previous research by systematically tracking SAID evolution, quantifying potential drops throughout different storm phases, and developing empirical models linking SAID characteristics to geomagnetic and substorm activity. Our coordinated multi-satellite approach shows two previously unreported findings: distinct two-phase SAID development patterns during storm progression and three-stage midlatitude trough evolution process linked to substorm activity. These findings represent the first systematic documentation of phase-dependent SAID development patterns and provide new quantitative insights into the coupling between ionospheric disturbances and geomagnetic storms. The subsequent sections detail our observational methodology (Sect. 2), present model framework and spatiotemporal analysis results (Sect. 3), discuss magnetosphere–ionosphere coupling implications (Sect. 4), and summarize key conclusions and future research directions (Sect. 5).

## Methodology

This study uses multi-point measurements observed by the DMSP satellite fleet, (consisting of three spacecraft: F16, F17, and F18) on June 1, 2013 in the Southern Hemisphere (negative MLAT)(Greenspan et al. 1986). We analyze ion drift velocities, plasma densities, and particle precipitation data collected during multiple orbital passes. We introduce a classification that divides the main phase into early and late phases to better characterize the temporal development of SAID features. We then detail our two-step criteria for discriminating between SAID and SAPS phenomena based on velocity thresholds and latitudinal width constraints. Finally, we present calculations for cross-field potential drops.

### DMSP satellite observations

Each DMSP satellite is equipped with multiple space environment instruments: the Special Sensors for Ionospheric Electrodynamics and Scintillation (SSIES), which includes the ion drift meter (IDM) and the retarding potential analyzer (RPA) (Greenspan et al. 1986); the precipitating energetic particle spectrometer (SSJ/4 and SSJ/5) (Hardy et al. 1984); and the Special Sensor Magnetometer (SSM) (Rich et al. 1985). DMSP satellites are in sun-synchronous orbits above the Earth at $\sim$850 km altitude with a $\sim$99$^{\circ }$ inclination, and their orbital periods are $\sim$100 min.

The initial and main phases of a geomagnetic storm (00 UT and 13 UT) were covered, and eight passes of the three DMSP satellites were analyzed. Each pass is labeled sequentially (e.g., the first pass of DMSP F16 is labeled F16$\_$1). We obtained DMSP data from CDAWeb (https://cdaweb.gsfc.nasa.gov) and Madrigal (http://cedar.openmadrigal.org) hosted by CEDAR. CDAWeb provides the data with Level 2 product of the RPA data (ion temperature, ram velocity, and chemical composition) and quality flags on several of the parameters. SSJ data were retrieved from Madrigal for F16 through F18, as some data were unavailable on CDAWeb.

The IDM sensor measures both vertical (V$_{z}$) and horizontal (V$_{y}$) ion drift components with a bimodal cadence; IDM switches between normal and slow modes depending on the total ion density (n$_{ion}$). In normal mode (n$_{ion}$ $\ge$ 2.1$\times$10$^{4}$ cm$^{-3}$), data are reported at a 1-s cadence for V$_{z}$ and V$_{y}$. In slow mode (n$_{ion}$ < 2.1$\times$10$^{4}$ cm$^{-3}$), the instrument alternates between V$_{z}$ and V$_{y}$ measurements every other second, resulting in a longer (2-s) cadence for V$_{y}$ and V$_{z}$. Thus, this paper uses both normal (1-s) and slow (2-s) cadence V$_{y}$ data. To represent the SAID accurately, IDM-reported quality flags are used to remove questionable data (flags $=$ caution, bad, or unknown).

### Storm-phase classification

This two-phase classification is motivated by the Type 2 geomagnetic storm development pattern described by Kamide et al. (1998), which exhibits distinct evolutionary stages during storm progression. We define the early main phase as showing initial SAID formation with pronounced equatorward movement, and the late main phase as characterized by SAID channel broadening, enhanced drift velocities, and deeper trough formation.

### SAID and SAPS discrimination criteria

To distinguish between SAID and SAPS, we apply two conditions. First, we use a velocity threshold of 900 m/s, considering that SAID typically exceeds 1 km/s. However, SAPS flows can also exceed 900 m/s between 1800 and 2000 MLT (Foster and Vo 2002). Therefore, meeting the velocity threshold alone does not definitively classify a flow channel as SAID. We apply the second condition that the latitudinal width of the flow channel should be less than 2$^{\circ }$ in MLAT, since SAID is generally more localized and confined to a narrower latitudinal band compared to SAPS (3$^{\circ }$–5$^{\circ }$) (Foster and Burke 2002).

### Cross-field potential drop calculation

To analyze the SAID structure quantitatively, we calculated the electric potential drop across each channel. Assuming that the observed structures are quasi-electrostatic, the potential drop ($\Phi$) is determined by integrating the electric field across the full width at half maximum (FWHM) of each SAID channel:

1 $$\Phi = \,\int {_{{{\mathrm{FWHM}}}} } \left| {{\mathbf{E}}(s)} \right|ds$$

where *E* is the electric field and *ds* is the differential distance element along the satellite. The quasi-electrostatic assumption is appropriate because SAID structure changes on timescales of minutes to hours, which are much longer than electromagnetic wave propagation times across the structure ($\sim 10^{-4}$ to $10^{-3}$ seconds for typical SAID widths of 50–200 km). It allows the electric field to be treated as the gradient of a scalar potential and ensures that the computed potential drop remains relatively insensitive to altitude variations (Anderson et al. 1993; Mishin et al. 2017).

In practical terms, we implemented this calculation using the following procedure:

We identified the FWHM of each flow channel by determining where the ion drift velocity ($v_y$) falls to half of its peak value. The electric field at each point is calculated using:

2 $$\begin{aligned} |{\vec {E}}| = |v_y|\,|{\vec {B}}|, \end{aligned}$$

where $v_y$ is the measured cross-track ion drift velocity (in m/s) and $|\vec {B}|$ is the directly measured magnetic field magnitude (in Tesla). Following Mishin (2023), we used the total field magnitude $|\vec {B}|$ rather than just the vertical component $B_z$ for two key reasons. First, the guiding-center drift formula $V_{\perp } = \vec {E} \times \vec {B}/B^2$ shows that drift speed scales with $1/|\vec {B}|$, so using the full field magnitude correctly accounts for the field geometry in all directions. Second, using $|\vec {B}|$ avoids sign and coordinate system ambiguities that could arise from local perturbations such as ring current depressions, which can create horizontal field components (Mishin and Puhl-Quinn 2007; Mishin et al. 2017).

The differential distance element is approximated using the satellite velocity and time step:

3 $$ds = \,\left| {\vec{v}_{{{\mathrm{sat}}}} } \right|\,dt$$

where $\vec {v}_{sat}$ is the satellite velocity ($\sim$7,500 m/s for the circular orbit with roughly constant speed) and *dt* is the time step between measurements.

The potential drop is then calculated by numerical integration over the FWHM region:

4 $$\Phi \approx \sum\limits_{{{i \in \mathrm{FWHM}}}} {\left| {v_{{y,i}} } \right|} \left| {{\mathrm{B}}_{{i}} } \right|\left| {\vec{v}_{{sat}} } \right|\Delta {\mathrm{t}}_{{i}}$$

## Results

Spatial and temporal variations of the SAID are analyzed by focusing on the main phase of the geomagnetic storm, and examining drift velocities, channel widths, and potential drops by comparing the early main phase and late main phase. We also analyzed the development of midlatitude troughs in accordance with substorm activity indicated by the AE index peaks. The ion and electron precipitation boundaries are examined in relation to the location of flow channels and their temporal changes throughout the storm development.

### Spatial distribution of SAID

**Fig. 1:**
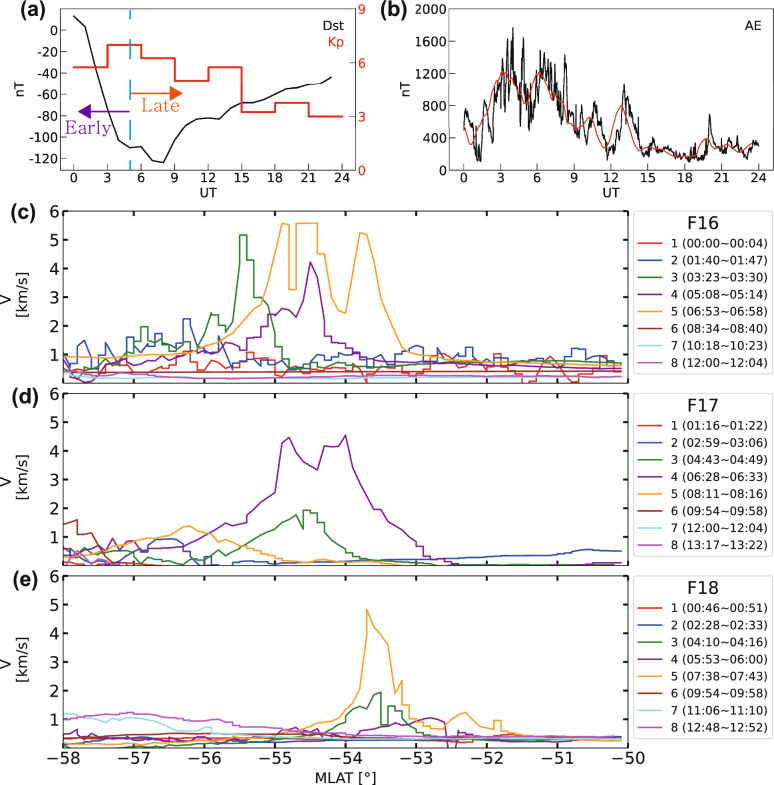
Event summary plots for 1 June 2013. **a** $K_p$ (red) and Dst (black) indices during the event from the Kyoto World Data Center for Geomagnetism, **b** AE index (black) during the event from the Kyoto World Data Center for Geomagnetism. The red line shows the simple moving average (SMA) of the AE data using a 60-min window, which smooths short-term fluctuations and highlights longer-term trends, (**c**−**e**) horizontal drifts of each pass for F16, F17, and F18 between −58$^{\circ }$ to −50$^{\circ }$ in MLAT. Periods of each interval are shown in the subsequent boxes (right)

**Fig. 2:**
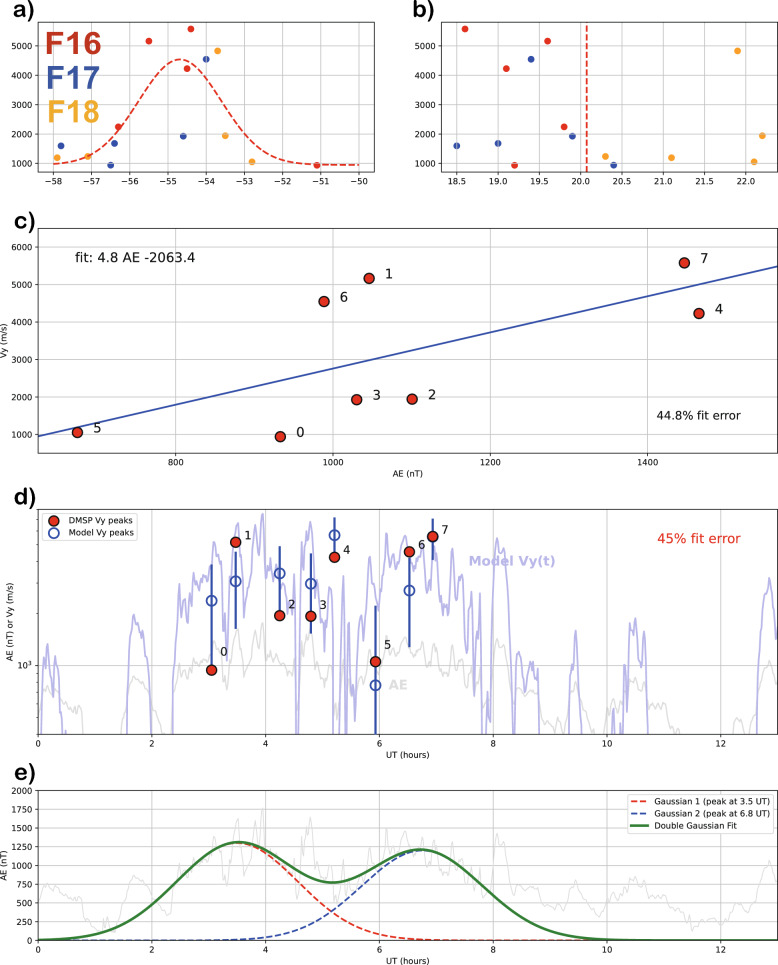
Comparison between observations and empirical SAID model predictions.** a** Vy versus MLAT with Gaussian fit (red line),** b** Vy versus MLT distribution with center (red dashed line),** c** Vy time series with AE correlation model,** d** Vy-AE scatter plot,** e** Double Gaussian AE fitting

Figure 1 summarizes the geomagnetic indices (Dst, $K_p$, and AE) and cross-track ion drift speed of three DMSP satellites on June 1, 2013. The Dst index decreased from 13 nT to its minimum of −124 nT (Fig. 1a) from 00 UT to 09 UT, and the storm progressed into the main phase. Based on the identification of Type 2 geomagnetic storm characteristics at $\sim$05 UT (see Sect. 2.2 for methodology), we define the period before $\sim$05 UT as the early main phase and after $\sim$05 UT as the late main phase. The $K_p$ index peaked at a value of 7 between 03 and 06 UT (Fig. 1a). The AE index (Fig. 1b) shows bimodal structures between 03 UT and 07 UT (first peak: $\sim$03 UT, second peak: $\sim$06 UT), as identified by the 60-minute moving average (red line). During the decrease in Dst and the peak in the $K_p$ index, fast westward ion flows were observed multiple times by each DMSP satellite in the Southern Hemisphere (Figs. 1c-1e). These fast westward flow channels exhibited typical SAID characteristics, including narrow latitudinal widths (<2 $^{\circ }$) and high drift speeds (>900 m/s). However, it is important to note that fast westward ion flows did not always appear coincidentally with decreasing Dst and elevated $K_p$ indices. For instance, at the onset of the Dst decrease around 00 UT, despite the $K_p$ index being $\sim$6, no significant westward flows were observed until around 01:30 UT. Similarly, no further prominent flows were observed following the second AE peak (after $\sim$07 UT), even as the Dst remained below −100 nT and $K_p$ was sustained at $\sim$6. This suggests that the generation and intensification of SAID are tied not only to the general conditions of a storm’s main phase but also to more immediate drivers, such as the substorm activity indicated by the AE index.

**Fig. 3:**
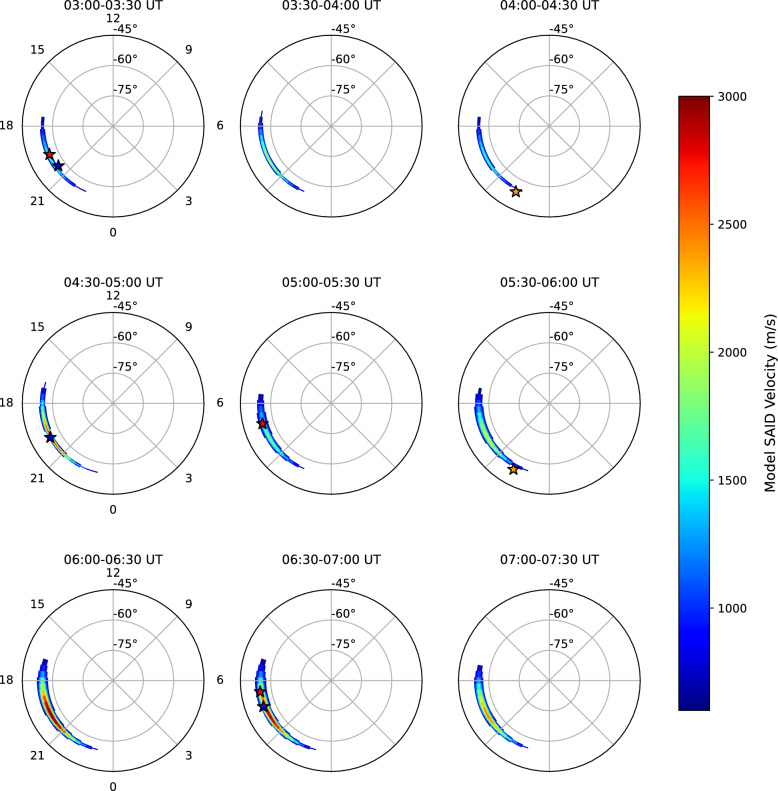
Time-dependent empirical SAID polar model predictions showing the spatial distribution of SAID velocities at 30-minute intervals from 03:00 to 07:30 UT. Each panel displays model-expressed velocity contours (color scale: 600–3000 m/s) overlaid with corresponding DMSP satellite observations (star markers) within each time window

**Fig. 4:**
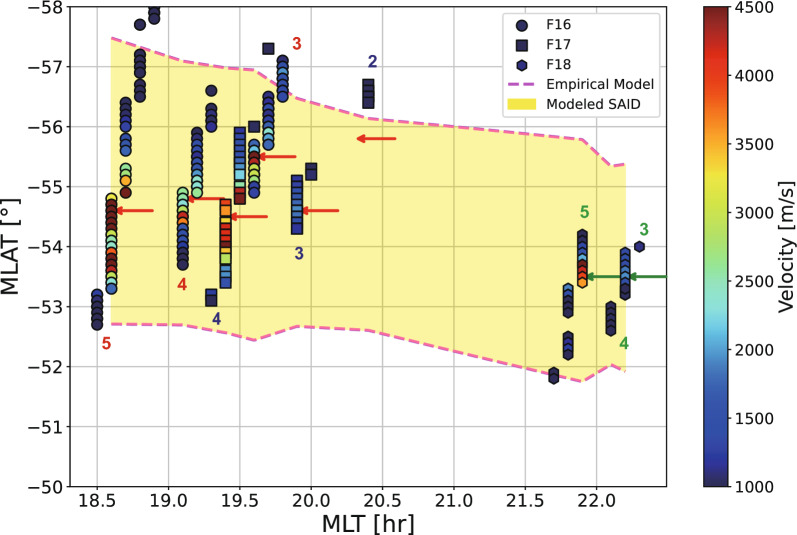
Spatial distribution of the SAID drift velocities observed by three satellites. Circular markers indicate F16 data, square markers indicate F17 data, and hexagon markers indicate F18 data. Numbers correspond to specific intervals of observation. The color bar represents drift velocities exceeding 900 m/s. Red arrows mark the locations of midlatitude density minima, while green arrows indicate the locations of cliff structures in ion density (Fig. 5)

### Ad hoc spatiotemporal empirical SAID model

The DMSP observations detailed in Sect. 3.1 provide scattered, snapshot-like measurements of the SAID structure. While these measurements are discrete in time and space, they exhibit an underlying coherence; the location and intensity of the observed flow channels appear to evolve systematically in response to substorm activity, as traced by the AE index. Therefore, we hypothesize that these scattered data represent a single, evolving event. The justification for this hypothesis is provided in the subsequent subsections (3.2.1 and 3.2.2), where we construct an empirical model to quantitatively reproduce the observed spatiotemporal evolution of the SAID. The purpose of this model is to test if a single, coherent framework—one that assumes a continuous structure whose intensity is modulated by the AE index—can approximately express/reproduce (within a factor of 2) the multiple DMSP observations:

5 $$\begin{aligned} V_{\text {SAID}}(AE, \lambda , \phi ) = V_{\text {base}}(\text {AE}) \times P_{\text {lat}}(\lambda ) \times P_{\text {mlt}}(\phi ), \end{aligned}$$

where *t* is time, $\lambda$ is MLAT, $\phi$ is MLT, and the function consists of a base velocity component $V_{\text {base}}$, a latitudinal profile $P_\text {lat}$, and a MLT profile $P_\text {mlt}$. It is worth noting that this model is built upon several assumptions, as detailed in the following subsections. Given these assumptions, the model’s performance is interpreted as being consistent with our hypothesis, but does not constitute a definitive proof.

#### Spatial characteristics of SAID

**Fig. 5:**
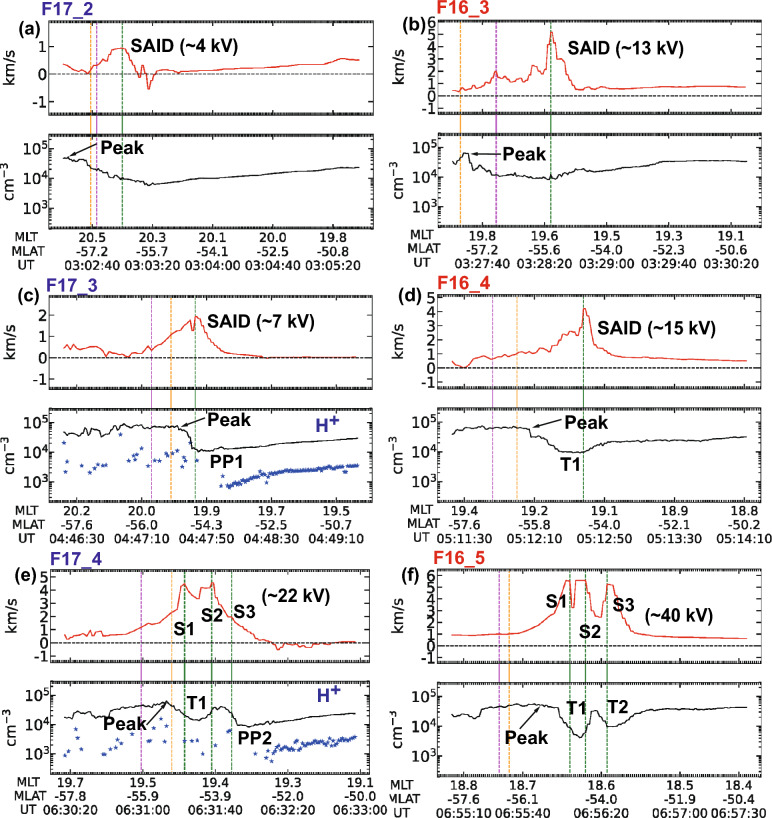
The spatiotemporal variations of the ion density and horizontal ion drift for F16 and F17 observations. (**a**–**)** present the intervals of F16 and F17 in chronological order, with each panel containing two subplots: the upper subplot shows the horizontal ion drifts (red line) and the lower subplot shows the ion density (black line). Blue stars represent H$^{+}$ density measurements. In the drift subplots, labels 'S1' through 'S3' mark individual flow channels within the SAID region with the estimated electric potential drop. Internal annotations in the density subplots identify key ionospheric features: the label 'Peak' denotes the auroral density maximum, 'T1' and 'T2' represent the primary and secondary midlatitude trough minima, and 'PP1' and 'PP2' indicate the identified ionospheric projections of the plasmapause. The vertical green dashed lines indicate the locations of the fastest horizontal drift. The vertical orange and magenta lines indicate the LLEP and LLIP locations, respectively, corresponding to the boundaries shown in Fig. 6

**Fig. 6:**
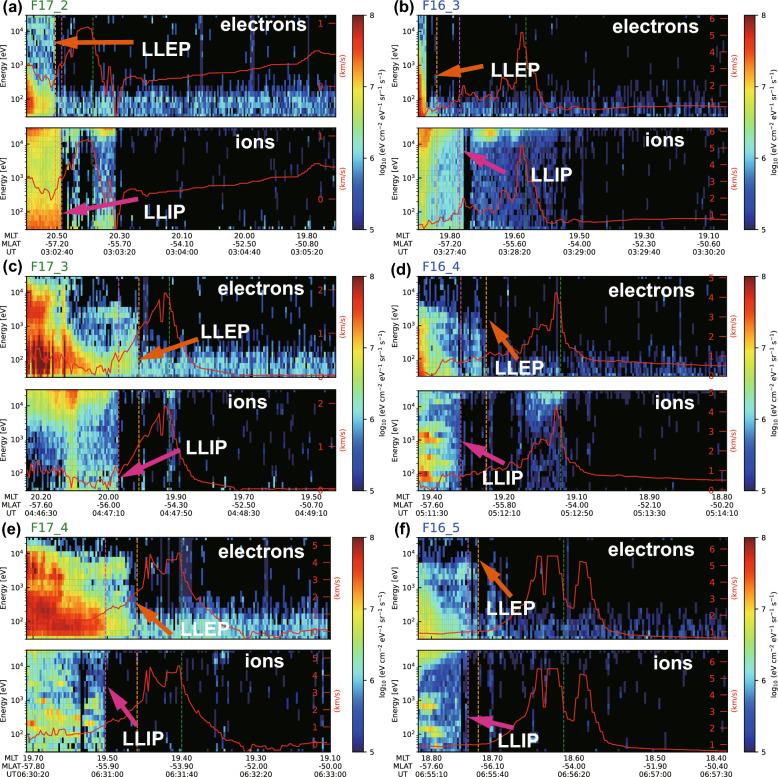
The spatiotemporal variations of electron and ion precipitating flux for F16 and F17 observations.** a**,** c**,** e** F17 results.** b**,** d**,** f** F16 results. The chronological order is the same with Fig. 5. The first panel and second panel in each section show the electron and ion precipitation data, respectively. LLEP denotes the low-latitude edge of electron precipitation and LLIP denotes the low-latitude edge of ion precipitation. The event periods used in this figure are identical to Fig. 3. The red line is the horizontal ion drift and the vertical green lines indicate the SAID peak drift velocity location which is the same as in Fig. 5

**Fig. 7:**
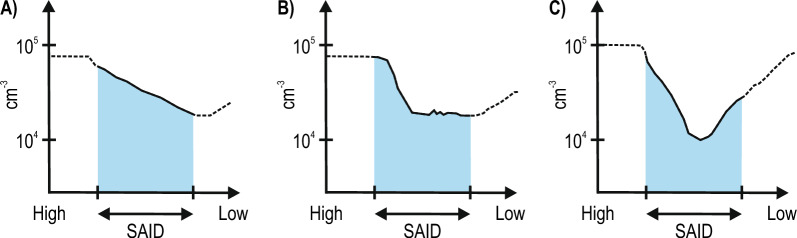
Schematic illustration of a three-stage development of the plasma density trough associated with a SAID. The blue shaded band marks the latitudinal extent of the SAID. The vertical axis presents plasma density in cm$^{-3}$ on a logarithmic scale from 10$^{4}$ to 10$^{5}$. The horizontal axis is MLAT, decreasing from high latitude on the left to low latitude on the right, and the arrows beneath each panel denote the width of the SAID. **A** Stage 1 represents the initial formation characterized by a mild density gradient. **B** Stage 2 illustrates the sharp density drop at the ionospheric projection of the plasmapause boundary. **C** Stage 3 depicts the deep trough

The fastest ion drift in each interval (Figs 1c-e) is indicated by colored points in Fig. 2. Some intervals were filtered out based on two criteria: drift speed (v$_{y}$ > 900 m/s) and MLAT (< −58$^{\circ }$). Following the SAID selection criteria (Sect. 2.3), 9 out of 24 intervals were removed to avoid contamination from auroral zone convection flows that can exhibit similar westward drift velocities.

Figure 2a presents the latitudinal distribution of the fast flow channels. The high velocity flows are concentrated at $\sim$−55$^{\circ }$ MLAT. We initially attempted to fit our data distribution using a single Gaussian model. However, since each flow channel was observed at different times and locations, the width derived from the Gaussian fitting was significantly larger than that of typical SAID. Thus, we developed a new latitudinal profile, $P_{\text {lat}}$, that models the narrow confinement of the SAID channel observed in Fig. 2a. We use a Gaussian function centered at a fixed magnetic latitude, with a time-dependent width that captures the observed evolution of the SAID channel structure:

6 $$\begin{aligned} P_{\text {lat}}(\lambda ) = \exp \left( -0.5 \left( \frac{\lambda - \lambda _{\text {center}}}{\lambda _{\text {width}}(t, \phi )} \right) ^2\right) , \end{aligned}$$

where $\lambda _{\text {center}}=-55^{\circ }$ is the fixed center latitude based on observations, $\lambda _{\text {width}}$ is the the time-dependent width parameter that transitions from narrow to broader channel structure at 5.0 UT:

7 $$\begin{aligned} \lambda _{\text {base\_width}}(t) = {\left\{ \begin{array}{ll} 0.8^{\circ } & \text {if } t < 5.0 \text { UT (early phase)} \\ 1.3^{\circ } & \text {if } t \ge 5.0 \text { UT (late phase)} \end{array}\right. }. \end{aligned}$$

This time-dependent width formulation is based on the observed FWHM evolution in Table 1, where the SAID channel exhibits a narrower width during the early storm development phase and broadens during the late main phase.

To better reproduce the observed spatial structure, we introduced a further refinement to this width parameter. While the formulation in Eq. (7) captures the temporal evolution, it does not account for the well-documented azimuthal asymmetry of the SAID channel, which is typically broader in the dusk sector and narrows toward midnight (Anderson et al. 2001). To incorporate this, the final latitudinal width, $\lambda _{\text {width}}(t,\phi )$, is calculated by modulating the time-dependent base width from Eq. (7) with a linear function of MLT:

8 $$\begin{aligned} \lambda _{\text {width}}(t,\phi ) = \lambda _{\text {base\_width}}(t) \times [1 - 0.2(\phi - \phi _{\text {ref}})], \end{aligned}$$

where $\phi$ is the MLT in hours and the reference MLT, $\phi _{\text {ref}}$, is 20 MLT. This modulation empirically reproduces the observed asymmetry; for instance, at 18:00 MLT, the channel width is expanded by a factor of $\sim 1.4$, whereas at midnight (24 MLT), it is narrowed by a factor of $\sim 0.2$, relative to the base width.

Figure 2b shows the distribution of the fast flow channels in Vy versus MLT. To mathematically represent this azimuthal profile, denoted as $P_{\text {mlt}}(\phi )$, we employed a Gaussian function. This approach follows established precedents in empirical modeling of the SAID/SAPS azimuthal structure. For instance, the foundational model of Goldstein et al. (2005) utilized a Fourier series to represent the broad azimuthal dependence of the average SAPS potential. Some other models have employed functionally similar forms such as a squared cosine profile for event-based studies (e.g., Califf et al. (2022)).

While functionally similar to these precedents, the Gaussian function is particularly advantageous for an ad hoc event model because its parameters directly correspond to the SAID channel’s key physical properties: the center MLT ($\phi _{\text {ref}}$) and the azimuthal width ($\Delta _{1/2}$). This direct parameterization allows these properties to be dynamically adjusted based on the observational data (Eq.(9)).

For the specific event in this study, the combined observations from the F16, F17, and F18 satellites span $\sim$3.5 h in MLT (Fig. 4). It is acknowledged that these multi-point in situ measurements alone cannot confirm the full azimuthal span of the SAID. Such confirmation typically requires supporting data from ground-based instruments, such as the Super Dual Auroral Radar Network (SuperDARN), or global imaging observations from like THEMIS (Time History of Events and Macroscale Interactions during Substorms) (Nishimura et al. 2020). Nevertheless, our observations are consistent with the hypothesis that they represent a single, evolving SAID structure with a significant longitudinal extent. While similar statistical studies on the azimuthal span of SAIDs are lacking, studies of the closely related SAPS have shown that they statistically span $\sim$3 h in MLT (Kunduri et al. 2017, 2018). Therefore, we assume our observation captures a part of a single SAID structure with a comparably wide azimuthal span.

Furthermore, our model is designed to capture the observed asymmetry in the channel’s structure. To achieve this, $\Delta _{1/2}$ is parameterized as a function of both MLT and peak velocity, calculated as:

9 $$\begin{aligned} \Delta _{1/2} = (a_0 + a_1(\phi - \phi _{\text {ref}})) \times \left( 1 + b_1\frac{V_{\text {current}} - V_{\text {ref}}}{V_{\text {ref}}}\right) , \end{aligned}$$

where the parameters are empirically determined. The MLT-dependent term, $a_0 + a_1(\phi -\phi _{ref})$, explicitly models the well-documented observation that the SAID/SAPS flow channel is typically broader in the dusk sector and narrows toward midnight (Anderson et al. 2001). The velocity-dependent term was included to empirically capture the specific width variations observed during this particular storm event. The base longitudinal half-width ($a_0$) was set to 2.0 h, yielding a full width that is consistent with the $\sim$3 h average MLT span reported by Kunduri et al. (2017, 2018).

This calculated width is then used within the Gaussian function to determine the final MLT profile:

10 $$\begin{aligned} P_{\text {mlt}}(\phi ) = \exp \left( -0.5 \left( \frac{|\delta _{\phi }|}{\Delta _{1/2}} \right) ^2\right) , \end{aligned}$$

where $|\delta _{\phi }|$ is the azimuthal distance from $\phi _{\text {ref}}$. Thus, our formulation for $P_{\text {mlt}}(\phi )$ represents an attempt to model the SAID azimuthal structure, capturing both its typical behavior and the specific features inferred from this event.

#### Temporal evolution and AE dependence

The temporal evolution of the model is governed by the function $V_{\text {base}}$, which we parameterize using the AE index as a proxy for substorm activity. To establish this relationship, we focused on the period of enhanced substorm activity (03–08 UT), as SAID is known to have a lifetime of approximately 30 min to 3 h and are closely linked to such activity (Anderson et al. 1991; Lejosne and Mozer 2017). However, the physical relationship between the magnetospheric driver (substorm energy injection) and the ionospheric response (SAID formation) is not instantaneous. Foundational observational studies have consistently shown that intense SAID events typically occur well after substorm onset, during the recovery phase, with a characteristic delay of approximately 30 min or more (e.g., Anderson et al. (1993)). Therefore, to construct a more physically robust and statistically sound model, we incorporate this known physical delay and include all available data points in our analysis. We performed a cross-correlation analysis by varying the time lag from 10 to 60 min and found that a delay of $\tau \approx 30$ minutes yielded the optimal fit, minimizing the RMS error. This result is in excellent agreement with previous statistical findings on M-I coupling timescales. The new linear relationship, based on all data points and a 30-minute time lag, is given by (Fig. 2d):

11 $$\begin{aligned} \text {V}_{base} = 4.8\times AE(t-30 min)-2063.4 \ (m/s). \end{aligned}$$

This empirical relationship achieved a root-mean-square (RMS) fit error of 45$\%$. We acknowledge that a direct theoretical basis for a strictly linear relationship between SAID velocity and the AE index is not well-established. We employ this linear fit as a first-order approximation for our ad hoc empirical model, intended to capture the primary trend within this specific event. As detailed in Appendix A, this approach is justified by the known linear trends between related geomagnetic indices and the SAPS, of which SAID is a more intense subset. The remaining 45$\%$ error likely reflects the influence of other unmodeled physical factors, such as local ionospheric conductivity, MLT, and the overall storm phase (e.g., Dst index), which could be explored in future, more comprehensive statistical studies.

For comparison and to characterize the bimodal AE structure observed during this storm, we performed a double-Gaussian fit to the AE index (Fig. 2e):

12 $$\begin{aligned} f(t) = A_1 \exp \left( -\frac{(t - \mu _1)^2}{2\sigma _1^2}\right) + A_2 \exp \left( -\frac{(t - \mu _2)^2}{2\sigma _2^2}\right) , \end{aligned}$$

where *t* is time, *A* is amplitude, $\mu$ is the peak time, and $\sigma$ is the standard deviation (width) of each Gaussian component. Within the 02–08 UT interval, this fit achieved approximately 20$\%$ error, successfully capturing the bimodal structure. However, as the Gaussian functions approach zero outside this range, the overall error increases substantially. Therefore, we use these parameters only to illustrate the bimodal features of the AE enhancement shown in Fig. 2e, not as the primary driver for our SAID model.

Figure 3 shows the output of a new time-dependent empirical SAID polar map model that expresses the spatial distribution of SAID in 30-minute intervals from 03:00 UT to 07:30 UT. The model is formulated following Eq. (5). The model incorporates a dynamic latitudinal center that captures the observed equatorward expansion during the early main phase (< 5.0 UT) followed by latitudinal stabilization in the late main phase ($\ge$ 5.0 UT). Additionally, the model features time-dependent channel widths that transition from narrow in the early phase to broader in the late phase. The series of nine polar plots shows the model’s performance by comparing its expressions to the DMSP observations (star markers) from the corresponding time windows. As time progresses from 03:00 UT, the model reproduces the intensification of SAID in response to the increasing average AE value, capturing both the spatial distribution and temporal evolution of the flow channel.

This agreement suggests that the empirical model may be capturing essential aspects of the primary dynamics of SAID evolution during this storm, quantitatively linking the development of the flow channel to the driving substorm activity. We believe that acceptable performance by the ad hoc model does lend support to the interpretation that our reported observations are from a single SAID whose spatiotemporal characteristics varied with time. The imperfect nature of the fit points the way toward a future study that uses a statistical ensemble of SAID flow observations.

Figure 4 shows a detailed examination of the flow channels identified during the substorm enhanced period (03–08 UT) in Fig. 2c. Peak drift speeds, along with the latitudinal locations and widths of the flow channels (calculated using the FWHM), are summarized in Table 1. The temporal separation between the observations made by F17 and F16 was approximately 25 min, with F17 passing first, followed closely by F16. Their spatial separation was about 0.8 h in MLT, and they were located at nearly identical latitudes. This close spatial and temporal proximity suggests that F16 and F17 observed the same SAID channel at slightly different locations and stages of evolution. Meanwhile, F18 detected comparable westward drift speeds (up to 4.8 km/s in the F18_5), although its observations were located 2–3 h away in MLT from those made by F16 and F17. Despite the 2–3 h MLT separation, the drift channels observed by F18 exhibited similar temporal evolution and decay patterns as those detected by F16 and F17. The magenta dashed curves represent the empirical model from (9), indicating predicted latitudinal boundaries and illustrating how the flow channel widens toward dusk sector and narrows toward midnight sector. These combined multi-satellite observations, spanning different MLT sectors and temporal intervals. It suggests that the observed flow channels represent manifestations of a single SAID with extended azimuthal continuity rather than multiple independent localized phenomena.

**Table 1:** The SAID location and width observed by F16, F17 and F18 showing basic characteristics of the event. This table presents the information of SAID observed from F16, F17, and F18 during the strongest disturbance periods

	F16$\_$3	F16$\_$4	F16$\_$5
*MLAT*($^{\circ }$)	− 55.5	− 54.5	− 54.4
*FWHM*($^{\circ }$)	0.8	1.6	0.8
*MLT*	19.6	19.1	18.6
$Peak \ time$	03:28:24	05:12:38	06:56:12
$SAID \ peak \ (km/s)$	5.58	4.23	5.58
*Potential*(*kV*)	12.94	15.32	40.20
	F17$\_$2	F17$\_$3	F17$\_$4
*MLAT*($^{\circ }$)	− 56.5	− 54.6	− 54.0
*FWHM*($^{\circ }$)	0.9	0.6	1.1
*MLT*	20.4	19.9	19.4
$Peak \ time$	03:03:00	04:47:43	06:31:38
$SAID \ peak \ (km/s)$	0.94	1.93	4.55
*Potential*(*kV*)	3.91	7.37	22.21
	F18$\_$3	F18$\_$4	F18$\_$5
*MLAT*($^{\circ }$)	$-$53.5	$-$52.8	$-$53.7
*FWHM*($^{\circ }$)	0.8	–	0.5
*MLT*	22.2	22.1	21.9
$Peak \ time$	04:14:51	05:58:53	07:42:26
$SAID \ peak \ (km/s)$	1.94	1.05	4.83

### Midlatitude trough structure and development

Figure 5 shows the variation of horizontal velocities and ion densities along the DMSP F16 and F17 tracks for the same intervals presented in Fig. 4. We identified the dynamic changes in the midlatitude trough structure throughout different storm phases.

Hereafter, we organized the F16 and F17 passes into three sets: (1) the first set (F17$\_$2 and F16$\_$3); (2) the second set (F17$\_$3 and F16$\_$4); and (3) the third set (F17$\_$4 and F16$\_$5) to track the evolution of the SAID event in three steps. In the first set, which corresponds to the early main phase, the flow peak in the F17$\_$2 was located at $-56.5^{\circ }$ MLAT with a FWHM of $0.9^{\circ }$ and a peak drift speed of 0.94 km/s. This relatively slow speed suggests that this flow represents the early stage of the SAID formation. The flow peak in the F16$\_$3 was observed at $-55.5^{\circ }$ MLAT with a FWHM of $0.8^{\circ }$, but with a much higher drift speed of 5.58 km/s. During this set, the first major AE enhancement period (centered around $\sim$ 03:05UT) occurred, and the Dst index was decreasing. Comparing these sequential observations, the flow peak position was observed $1^{\circ }$ more equatorward in the F16$\_$3 than in the F17$\_$2, with minimal change in channel width (from $0.9^{\circ }$ to $0.8^{\circ }$). In the second set, which corresponds to the main phase of the storm ($\sim$80 min after the first set), the flow peak in the F17$\_$3 was observed at $-54.6^{\circ }$ MLAT, with a FWHM of $0.6^{\circ }$ and a peak drift speed of 1.93 km/s. In the F16$\_$4, the flow peak was observed at $-54.5^{\circ }$ MLAT, with a wider FWHM of $1.6^{\circ }$ and a drift speed of 4.23 km/s. Compared to the first set, the peak of the flow channel was detected about $1^{\circ }$ farther equatorward than in the initial observations. In the F16 observations, the channel width increased from $0.8^{\circ }$ to $1.6^{\circ }$. While the flow peak speed decreased from the previous F16 observation, the AE index decreased from its peak and the Dst index indicated that the storm had transitioned from the early main phase to the late main phase. In the final set ($\sim$ 80 min after the second set), the flow peak in the F17$\_$4 was located at $-54.0^{\circ }$ MLAT with a FWHM of $1.1^{\circ }$ and a peak drift speed of 4.55 km/s. The flow peak in the F16$\_$5 was located at $-54.4^{\circ }$ MLAT, with a FWHM of $0.8^{\circ }$ and a higher drift speed of 5.58 km/s. Since the second set, the F17 channel width broadened from $0.6^{\circ }$ to $1.1^{\circ }$, while the F16 channel narrowed from $1.6^{\circ }$ to $0.8^{\circ }$, and overall flow speeds increased in both satellites.

During the early main phase of the storm after 03 UT (Fig. 1a) and the first peak in the AE index ($\sim$03:05 UT) (Fig. 1b), we observed initial SAID formation without a well-defined trough structure, showing only a gradual density gradient. In interval F17$\_$2, the density gradient was mild despite the presence of a flow channel. Similarly, in F16$\_$3, although the westward flow reached a maximum speed of $\sim$5 km/s, the density gradient remained relatively flat. This pattern suggests that during early SAID formation, density gradients develop gradually while the midlatitude trough is still forming.

As the storm progressed to the main phase (Fig. 5c-d), a notable change occurred approximately 80 min after the initial observations. A sharp density gradient (marked as PP1) emerged near the SAID peak in F17$\_$3, indicating the ionospheric projection of the plasmapause. This gradient coincided with the flow channel peak in both latitude and time, appearing after the flow peak observed in F16$\_$3 (Fig. 6c). Similar development of cliff structures was reported by Anderson et al. (2008), who identified the ionospheric projection of the plasmapause using DMSP measurements of the H$^+$ density and EUV images of the plasmasphere. The H$^{+}$ density measurements showed a density drop by a factor of $\sim$10 near the steep structure of the midlatitude trough (Fig. 6b, marked by blue stars), indicating the ionospheric projection of the plasmapause (PP1).

By the time F16$\_$4 passed through the SAID, this steep gradient had evolved into a clear trough minimum (T1, in Fig. 5d), demonstrating the rapid development of trough structures during this phase. Comparing sequential observations from Figs. 6a-5c, the high-density region was detected progressively more equatorward, with a steep density gradient forming near the SAID. It should be noted that these observed positional differences do not necessarily indicate actual equatorward plasma transport, but rather reflect temporal changes in the spatial distribution of the density structure between measurements.

In the late main phase (panels e-f), the variations became more complex with multiple flow channels developing simultaneously. Double flow peaks (labeled S1 and S2) were observed at higher latitudes, while another flow peak (labeled S3) appeared at lower latitudes, exhibiting a slower speed compared to S1 and S2. Although the S1 and S2 peaks in F17$\_$4 and F16$\_$5 data had similar structures, the S3 peak in F17$\_$4 (with a speed of approximately 2 km/s) evolved into a faster flow channel in F16$\_$5, reaching a maximum speed of around 5 km/s.

The midlatitude trough (T1) remained at the same MLAT for 80 min, corresponding to S1 and S2 peaks (Fig. 6e). A new steep density gradient (PP2) appeared at lower latitudes than T1, collocated with the S3 peak. After 25 min, in F16 5, T1 deepened further (Fig. 6f), while another deep trough (T2) was observed near the newly developed cliff structure (PP2). The formation of the second trough structure coincided with the second major AE enhancement period (centered around $\sim$06:10 UT) (Fig. 1b). The temporal correlation between the emergence of multiple trough structures and AE index peaks demonstrates the close relationship between substorm activity, SAID intensification, and trough formation.

The calculated potential drops (labeled in Figs. 5a-f) showed distinct patterns across early and late main phases. During the early main phase (F17_2, F16_3, and F17_3), potential drops ranged from 3.91 kV to 12.94 kV. In contrast, during the late main phase (F16_4, F17_4, and F16_5), we observed much higher potential values ranging from 15.32 kV to 40.20 kV.

### Precipitation boundary variations associated with storm phases

The spatial relationship between the SAID, midlatitude trough, and precipitation boundaries showed systematic variations throughout all observations. Figure 6 shows the storm-phase changes in electron and ion precipitation flux in the form of energy and time spectrograms for F16 and F17, corresponding to the same intervals shown in Fig. 5. We identified both precipitation boundaries by electron and ion flux cutoffs: the low-latitude edge of electron precipitation (LLEP) and the low-latitude edge of ion precipitation (LLIP). The detailed information on the SAID, midlatitude trough, and precipitation boundaries is summarized in Table 2. In the first set (Figs. 6a-6b), we observed that the LLIP and LLEP initially coincided in F17$\_$2 with a minimal separation of only 0.2$^{\circ }$. After 25 min, this separation increased to 0.9$^{\circ }$, with the LLIP extending further equatorward than the LLEP. During this period, the SAID peak remained equatorward of both precipitation boundaries. Higher ion densities were observed near the LLEP, with the ion density peak at $-$57.3$^{\circ }$ MLAT in F17$\_$2 and $-$57.7$^{\circ }$ MLAT in F16$\_$3. In the second set (Figs. 6c-6d), a notable reversal occurred in the boundary configuration. The LLEP was now positioned at lower latitudes than the LLIP, with this reversed separation being more pronounced in F16$\_$4. The ion density peak in F16$\_$4 was observed at $-56.3^{\circ }$ MLAT at 19.3 MLT, compared to F17$\_$3 where it was detected at $-55.2^{\circ }$ MLAT at 20.0 MLT, indicating an equatorward expansion in the observed structure. Concurrent with these boundary shifts, the midlatitude trough became more defined (as shown in Figure. 5c-5d). In the final set (Figs. 6e-6f), as multiple flow channels developed (labeled S1, S2, and S3 in Figure. 5e-5f), both precipitation boundaries maintained their configuration with the LLEP positioned equatorward of the LLIP. The ion density peak was observed at progressively lower latitudes, reaching $-$55.1$^{\circ }$ MLAT in F17 and $-$56.4$^{\circ }$ MLAT in F16.

**Table 2:** The locations of minimum density, SAID, LLEP, and LLIP, as shown in Fig. 6 and 6

	UT	MLAT	MLT	UT	MLAT	MLT
	F16$\_3$			F17$\_2$		
$Density \ minimum$	–	–	–	–	–	–
*SAID*	03:28:24	− 55.5	19.6	03:03:00	− 56.5	20.4
*LLEP*	03:27:26	− 57.7	19.9	03:02:39	− 57.3	20.5
*LLIP*	03:27:49	− 56.8	19.8	03:02:43	− 57.1	20.5
	F16$\_4$			F17$\_3$		
$Density \ minimum$	05:12:33 (T1)	− 54.8	19.1	04:47:40 (PP1)	− 54.6	19.9
*SAID*	05:12:38	− 54.5	19.1	04:47:40	− 54.6	19.9
*LLEP*	05:12:00	− 56.3	19.3	04:47:28	− 55.2	20.0
*LLIP*	05:11:46	− 56.9	19.3	04:47:16	− 55.7	20.0
	F16$\_5$			F17$\_4$		
$Density \ minimum$	06:56:09 (T1)	−54.6	18.6	06:31:28 (T1)	− 54.5	19.4
	06:56:23 (T2)	− 53.8	18.6	06:31:52 (PP2)	− 53.3	19.3
*SAID*	06:56:04 (S1)	− 54.8	18.6	06:31:23 (S1)	− 54.8	19.5
	06:56:12 (S2)	− 54.4	18.6	06:31:38 (S2)	− 54.0	19.4
	06:56:23 (S3)	− 53.8	18.6	06:31:49 (S3)	− 53.5	19.4
*LLEP*	06:55:33	− 56.4	18.8	06:31:16	− 55.1	19.5
*LLIP*	06:55:28	− 56.7	18.8	06:30:59	− 55.9	19.5

$^a$ Trough (T); plasmapause (PP)

$^b$ Low latitude edge of ion (LLIP) and electron precipitation (LLEP)

The relative positions of the LLIP and LLEP precipitation boundaries showed a distinct evolution. Initially, the two boundaries were nearly co-located (F17$\_$2, F16$\_$3), after which the LLIP extended further equatorward than the LLEP (F16$\_$4). A persistent reversed configuration was observed during the later storm phases, where the LLEP was located equatorward of the LLIP (F16$\_$5, F17$\_$3, F17$\_$4).

Despite this dynamic evolution of the boundary configuration, two key spatial relationships remained consistent across all observations: first, every identified flow channel, including the multiple peaks observed in the late main phase, was located equatorward of the entire precipitation region defined by both the LLEP and the LLIP. Second, the ion density peak was found near the poleward edge of this precipitation zone, closely associated with the LLEP and LLIP locations. The observations show that while the internal configuration of the precipitation boundaries evolved dynamically as the storm progressed, the spatial relationship between the SAID channel and the overall precipitation zone remained stable.

## Discussion

Our multi-satellite observations show distinct spatiotemporal variations of the SAID and midlatitude trough during geomagnetic storms and substorm events. Based on these results, we discuss the two-phase variations of the SAID and development of the midlatitude trough and their association with DSAID. In addition, we discuss and suggest the ideal configuration of a satellite constellation for further understanding the spatiotemporal properties of SAIDs and midlatitude troughs for future observations and missions.

### Two-phase SAID evolution

Our multi-satellite observations captured a two-stage development of the SAID structure during the main phase of the storm. Stage 1 (early main phase) was characterized by a consistent equatorward progression of the flow channel, with moderate potential drops (3.91 to 12.94 kV). Stage 2 (late main phase) was marked by a cessation of this equatorward motion and a pronounced intensification of the electric field, with potential drops increasing to 15.32-$-$40.20 kV. This observed evolution is expressed by the ad hoc empirical model presented in Sect. 3.2. This progression also shares characteristics with the ’Type 2’ storm development pattern described by (Kamide et al. 1998) and is consistent with the dynamic evolution of SAPS predicted by global circulation models (Lin et al. 2019).

Stage 1 appears to represent the initial formation and earthward expansion of the partial ring current. During this period, the magnetospheric structures that define the subauroral region shift earthward as a whole. The ion and electron precipitation boundaries move equatorward in concert, maintaining a relatively wide separation. The resulting subauroral electric field is correspondingly broad, characteristic of a SAPS, which is consistent with the moderate potential drops observed. The transition to Stage 2 and the associated intensification of the electric field coincide with a key reconfiguration of the precipitation boundaries. During this stage, we observed a reversal of the typical boundary configuration, wherein the LLEP boundary was located equatorward of the LLIP boundary (Fig. 6, Table 2). This configuration departs from the conventional evening-sector pattern (Gussenhoven et al. 1987). We interpret this boundary reconfiguration as a consequence of substorm particle injection occurring preferentially in the pre-midnight sector, a mechanism consistent with recent studies (Nishimura et al. 2020, 2020). The location of the particle injection source is an important parameter for the subsequent structuring of the inner magnetosphere. As noted by Nishimura et al. (2020), an injection source shifted toward dusk provides a direct supply of a new electron population into the pre-midnight region. For the keV particles relevant to our study, the dominant earthward transport mechanism is the E$\times$B drift from the large-scale convection electric field, which acts on both electrons and ions. The distinction arises from the location of the particle source; the duskward-shifted injection preferentially populates the pre-midnight sector with these newly supplied electrons. This population is then transported earthward via E$\times$B drift, leading to an equatorward displacement of the LLEP in this local time sector. The LLIP, largely defined by the pre-existing, westward-drifting ring current, does not experience a comparable influx of particles from a nearby source in this sector and thus appears relatively stable.

In our observations, this boundary reversal and the SAID are located around 18–20 MLT. This location is further toward dusk than the $\sim$21 MLT cases discussed by Nishimura et al. (2020), but the underlying mechanism appears to be the same. It is plausible that the intense main phase of this geomagnetic storm produced a partial ring current that was more extended toward dusk. Such a configuration would shift the region of strongest pressure gradients, and thus the location of preferential particle injection, further into the dusk sector. Furthermore, this interpretation is consistent with previous observations showing that the electron precipitation boundary can extend equatorward of the ion boundary in the pre-midnight sector during substorms. Fujii et al. (1990) noted that with approaching midnight, the equatorward boundary of central plasma sheet (CPS) electron precipitation extends toward and eventually equatorward of that of the Region 2 current, which is closely associated with the ion boundary. These findings suggest that the boundary reconfiguration we observed is a fundamental aspect of the magnetosphere’s response to strong, dynamic driving.

The consequence of this process is a narrowing of the latitudinal gap between the ion and electron boundaries. As explained by Nishimura et al. (2020), it is this narrow, low-conductance channel that facilitates the development of an intense, localized poleward electric field. This confinement results in the formation of a narrow and fast SAID channel, as opposed to a broader SAPS. The transition from the moderate, progressing SAPS of Stage 1 to the intense, latitudinally stable SAID of Stage 2 can thus be understood as the direct ionospheric result of this injection-driven boundary reconfiguration. Our event is therefore consistent with this process and suggests its applicability extends further toward dusk during highly active conditions.

### Development of the midlatitude troughs and their association with DSAID

To our knowledge, multiple satellite observations indicate that the midlatitude trough proceeds through three stages that have not been documented in earlier work. Figure 7 presents a schematic illustration of this three-stage development, sketched from passes F17_2, F17_3, and F16_4: (A) formation of a mild density gradient, (B) appearance of a steep density drop near the plasmapause cutoff mapped to the satellite altitude, and (C) establishment of a persistent ionization trough. This three-stage development reflects the interplay between magnetospheric and ionospheric processes during storm progression.

The first stage is characterized by a mild density gradient as observed in pass F17$\_$2 (Fig. 5a). This feature is consistent with the classic formation mechanism for the dusk-sector midlatitude trough. In this mechanism plasma flux tubes stagnate in a region of opposing flows. The slow eastward co-rotation of the plasmasphere is opposed by the faster westward return flow of the high-latitude convection pattern (Spiro et al. 1978). Plasma in this stagnation region remains in darkness for an extended time without solar EUV photoionization. This allows normal recombination processes to slowly deplete the ion density. The result is a broad shallow trough that serves as the background condition for subsequent development (Rodger et al. 1992). This interpretation also aligns with previous findings that a midlatitude trough is often present prior to SAID formation, with the SAID then acting to deepen the poleward extent of the pre-existing structure (Figueiredo et al. 2004).

The second stage involves the formation of a sharp density drop at the ionospheric projection of the plasmapause. This feature is seen in pass F17$\_$3 (PP1 in Fig. 5c). This steep gradient is the direct result of plasma evacuation driven by the intensified SAID channel. Our observations show that plasma inside the plasmasphere co-rotates while plasma in the SAID channel drifts rapidly westward. The narrow high-speed westward flow ($\vec {v}_{SAID} \gg 1$ km/s) creates an extreme velocity shear against the slow co-rotating plasmaspheric plasma. This shear flow rapidly transports or "evacuates" lower-density plasma from the nightside. The flow effectively carves out the plasmapause boundary and steepens the density gradient into a "cliff-like" structure (Anderson et al. 2001). The precise co-location of this sharp gradient with the SAID velocity peak provides strong evidence for this transport-dominated mechanism.

The third stage is the establishment of a deep and persistent ionization trough. We observe this feature evolving from the steep gradient (PP1) to a distinct minimum (T1) between the F17$\_$3 and F16$\_$4 passes (Figs. 5c-d). This deepening is a consequence of enhanced chemical recombination driven by ion-neutral frictional heating within the SAID channel. The high relative drift between ions and the neutral thermosphere elevates the ion temperature ($T_i$) significantly (Moffett et al. 1992). This reaction is the primary rate-limiting loss process for the dominant F-region ion $O^{+}$ (Rodger et al. 1992). The rapid conversion of long-lived $O^{+}$ to short-lived molecular ions results in an accelerated net loss of plasma. The $\sim$25-minute temporal separation between the F17$\_$3 and F16$\_$4 observations captures this time-integrated chemical process. It shows the trough evolving from a transport-defined boundary to a chemically depleted minimum. This mechanism was first detailed in modeling studies by Schunk et al. (1976) and has been confirmed in numerous subsequent studies (e.g., Anderson et al. (1991))

The temporal correlation between major AE enhancement periods and the formation of new flow channels and troughs observed in our study shows an important causal relationship in SAID development. We observed multiple flow peaks, corresponding to DSAID described by He et al. (2016) and Wei et al. (2019). Building on those previous studies, our multi-point observations provide additional insight into the development of the midlatitude trough associated with DSAID. The emergence of separate flow peaks and troughs suggests that multiple flow channels can develop independently, especially when the initial flow channel persists until another forms. The first major AE enhancement ($\sim$03:05 UT) coincided with the initial formation of the SAID and the beginning of the midlatitude trough development, while the second major enhancement period ($\sim$06:10 UT) was followed by the emergence of a secondary flow channel and trough. This suggests that substorm injection processes, as indicated by sustained AE enhancements, may trigger the formation of new flow channels and midlatitude troughs. This substantiates the theoretical pathway proposed by Anderson et al. (1991) and extends it. Our observations that multiple substorm injections during a single storm can create independent SAID structures that coexist spatially and temporally are consistent with mechanisms such as the formation of multiple R2-FACs from recurrent injections (Wei et al. 2019) or multiple FAC sheets from plasma sheet density fluctuations (Ebihara et al. 2005)

### Implications for future observations and missions

In this study, we analyzed multi-point DMSP data to elucidate the spatiotemporal evolution of the SAID event, identifying a two-phase development pattern and a three-stage progression of the associated midlatitude trough. However, we encountered inherent observational limitations due to the current satellite configuration. A notable example is the ambiguity in our analysis of the F18 satellite data; its 2–3 h MLT separation from the F16 and F17 spacecraft made it difficult to determine whether F18 observed a spatially continuous structure or a separate, independent event. Although F18 was separated by approximately 2–3 h in MLT from F16 and F17, the similar temporal evolution of the flow channel, including its intensification and decay, suggests that it could be considered part of the same SAID structure. However, to substantiate this interpretation with higher confidence, supporting observations would be required, such as ground-based measurements to image the full longitudinal span of the flow channel (Nishimura et al. 2020), or denser multi-point in situ measurements. This limitation underscores the necessity for a future satellite constellation specifically designed to better characterize SAID structures.

To resolve such limitations for future research, we have estimated the required number of satellites and their temporal separation for an ideal constellation mission. Spatially, SAID statistically occurs within 18–24 h in MLT (He et al. 2014), while a single SAPS/SAID event can span approximately 3 h in MLT (Kunduri et al. 2017, 2018). As shown by our F18 data case, the uncertainty in our F18 data interpretation arises from the substantial 2–3 h MLT gap between observations, which underscores the fundamental difficulty of resolving a $\sim$3 hr spanning scale structure with widely separated measurements. This suggests an optimal inter-satellite separation of approximately 1 hr in MLT. Temporally, the rapid evolution of these events must also be captured. Given that SAID can have lifetimes as short as $\Delta t_{SAID}$ $\approx$ 30 min Anderson et al. (1991); Lejosne and Mozer (2017), the Nyquist–Shannon sampling theorem provides the quantitative basis for the required measurement cadence. The theorem stipulates that the sampling interval should be no greater than half the minimum lifetime to unambiguously resolve the temporal change. This dictates a required revisit time of 15 min or less (i.e., 30 min/2 = 15 min). Therefore, a future mission consisting of a six-satellite constellation, appropriately phased to meet both these spatial and temporal requirements, would provide the measurements needed to build upon the findings of this study and enable a more definitive investigation into the spatiotemporal evolution of SAID.

A comprehensive statistical analysis integrating both in situ and ground-based measurements remains essential to precisely constrain $\Delta \text {MLT}_{\text {SAID}}$ and $\Delta t_{\text {SAID}}$, informing future constellation missions of the optimal configuration. Our analysis suggests that a minimum of six satellites, strategically positioned and phased to meet both spatial and temporal sampling requirements, would be ideal to further resolves the temporal and spatial resolutions of SAPS/SAID phenomena beyond what we could study with three DMSP satellites.

## Conclusions

This study has examined the SAID event and associated midlatitude troughs during the June 1, 2013 geomagnetic storm using observations from three DMSP satellites in the Southern Hemisphere. Building upon the multi-satellite SAID observation methodologies (Anderson et al. 2001; Mishin et al. 2017), this research further advanced our understanding by developing empirical models that quantitatively reproduce the observed SAID, thus elucidating the relationship between SAID development and geomagnetic activity. Through multi-point measurements and the empirical models, we identified two key findings regarding the spatiotemporal development of the SAID and midlatitude troughs. First, we identified a two-phase development pattern of the SAID across the main phase. During the early main phase, the SAID showed prominent equatorward expansion with minimal width variations and moderate potential drops (3.91$-$12.94 kV). In contrast, the late main phase featured latitudinal stabilization with notable width variations and substantially enhanced potential drops (15.32$-$40.20 kV), representing more than a doubling of electric field intensity. This transition from spatial expansion to electric field intensification suggests an underlying change in the magnetospheric drivers, possibly indicating the progression from initial particle injection to the establishment of stronger current systems during the storm’s development. Second, we identified that the midlatitude trough developed through three stages: (1) mild density gradient formation associated with the initial AE increase; (2) sharp density drop at the plasmapause boundary after the first AE decrease; and (3) persistent deep trough formation after the first AE peak (at $\sim$03 UT) that continued throughout the second AE peak (at $\sim$06 UT). The temporal correlation between major AE enhancements and the formation of new trough structures implies that each sustained period of substorm activity can independently trigger the development of distinct flow channels. Our empirical model predicts the intensification in SAID velocities and positions in response to AE variations, demonstrating the causal relationship through which substorm injections drive the formation of multiple flow channels and associated trough structures. This observation extends previous DSAID studies by showing how multiple trough structures form independently in response to separate substorm events during a single storm.

These findings provide new quantitative insights into the temporal evolution patterns of SAID and midlatitude troughs, advancing our understanding of magnetosphere–ionosphere coupling during geomagnetic storms. Future multi-platform observations combining magnetospheric and ionospheric measurements, along with more sophisticated modeling efforts, will further elucidate the complex interplay between SAIDs and midlatitude troughs.

## Figures and Tables

**Fig. 8:**
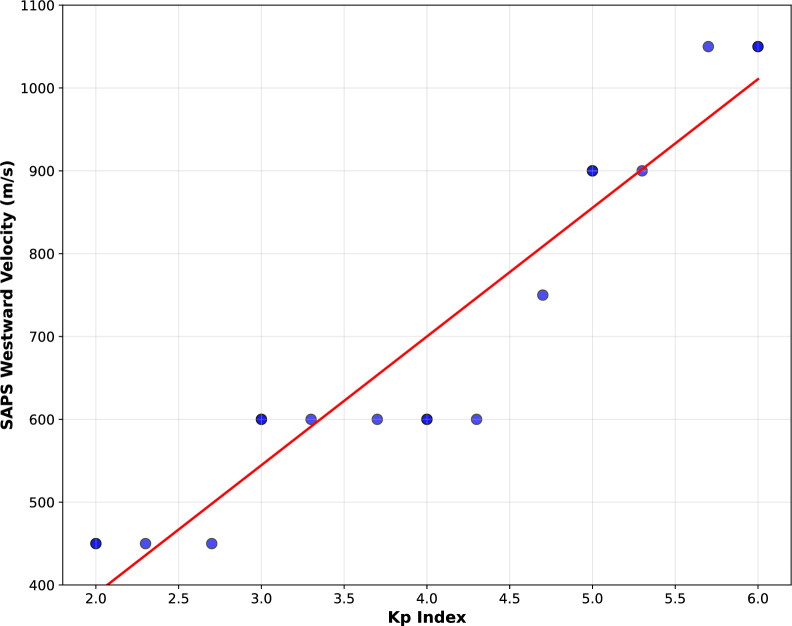
Linear relationship between SAPS westward velocity and the Kp index. The data points (blue circles) were derived by digitizing the contour plot from the statistical study by Foster and Vo (2002). The red line represents the linear regression fit, demonstrating a positive linear trend between average SAPS velocity and $K_p$

**Fig. 9:**
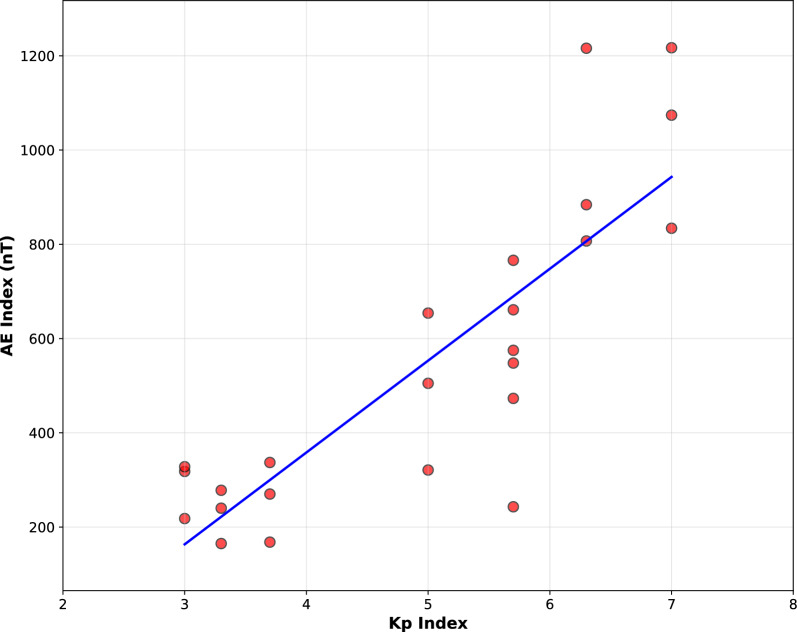
Linear correlation between AE and $K_p$ indices for the June 1, 2013 event. Scatter plot of hourly AE index values versus the corresponding 3-hour $K_p$ index values for the 24-hour period of the storm event. The data points (red circles) show a clear positive trend, and the blue line represents the linear regression fit, confirming a strong linear relationship between the two indices for this specific day

## Data Availability

Dst, $K_p$, and *AE* indices were provided by the World Data Center for Geomagnetism (WDC-C2) website, maintained by Kyoto University, Japan. The DMSP data were obtained from CDAWeb (https://cdaweb.gsfc.nasa.gov/) and Madrigal (http://cedar.openmadrigal.org/list)

## Appendix A: Justification for the linear relationship assumption

### A.1: Linearity between SAPS velocity and $K_p$ index

To establish a baseline for the relationship between subauroral drift velocity and a global geomagnetic activity index, we analyzed the statistical results presented by Foster and Vo (2002). Their study provides a contour plot showing the average magnitude of SAPS westward velocity as a function of both $K_p$ index and MLT. To derive a one-dimensional relationship from this two-dimensional plot, we extracted velocity values along a single, representative slice at a fixed MLT of 20 hrs, a local time where SAID/SAPS events are commonly observed. The resulting scatter plot of SAPS velocity versus $K_p$ index, shown in Fig. 8, demonstrates a clear linear trend. This indicates that, on a statistical basis, the average magnitude of SAPS velocity increases linearly with the $K_p$ index.

### A.2 Linearity between AE and $K_p$ indices for the 2013 June 1 event

To connect the general statistical behavior of SAPS to our specific event and chosen activity index, we examined the relationship between the $K_p$ and AE indices for the day of our event, 2013 June 1. As shown in Fig. 9, we plotted the hrly AE index values against the corresponding 3-hr $K_p$ index values for the entire 24-hour period. The analysis reveals a strong linear correlation between the two indices for this specific day. This demonstrates that for our event, the AE index serves as a reliable linear proxy for the planetary geomagnetic activity represented by $K_p$.

### A.3 Synthesis and conclusion

The evidence presented in this appendix forms a logical basis for our modeling approach. The statistical work of Foster and Vo (2002) establishes a linear relationship between SAPS velocity and $K_p$ (A.1), and our analysis confirms a linear relationship between $K_p$ and AE for the specific storm event under study (A.2). In addition to this inference, a recent case study by (Zhang et al. 2021) provides direct observational support, reporting good linear correlations between SAPS velocity and the AE index.

Given that SAID is often understood to be a more localized and intense manifestation of the SAPS, our assumption of a linear relationship between SAID velocity and the AE index serves as a justified and appropriate first-order approximation for our event-specific empirical model.
